# Supplementary material for: Strengthening access to and confidence in COVID-19 vaccines among equity-deserving populations across Canada: An exploratory qualitative study
Source: PLoS One. 2026 Apr 27;21(4):e0301953. doi: 10.1371/journal.pone.0301953 (PMC13120697; doi:10.1371/journal.pone.0301953)
Supplement: S1 Table — (DOCX) [file pone.0301953.s001.docx]

**S1 Table. Consolidated criteria for reporting qualitative studies (COREQ): 32-item checklist**

| **No. Item** | **Guide questions/description** | **Remarks** | **Reported on Page #** |
| --- | --- | --- | --- |
| **Domain 1: Research team and reﬂexivity** | | | |
| ***Personal Characteristics*** | | | |
| 1. Interviewer/facilitator | Which author/s conducted the interview or focus group? | Semi-structured interviews were led by two research team members (MS and VA) and averaged about 1 hour (interview length ranged from 33 to 85 minutes). | Under Materials and Methods |
| 2. Credentials | What were the researcher’s credentials? E.g. PhD, MD | KB – MA, BSc.  MO – MSc., BSc.  VA - MSc., BSc.  MS – MPH, BSc.  CD – MSc., BSc.  DMV – MSc., BKin  SA – PhD, MSc.,  AA – PhD, MSc., BSc.  SF - PhD, MPH, BSc. |  |
| 3. Occupation | What was their occupation at the time of the study? | MO, VA, MS and CD are PhD students in Epidemiology at the University of Toronto. KB was a PhD student in Health Services Research at the University of Toronto and DMV was a Master’s student at the University of Toronto. All students were members of the Implementation Science training program at Dalla Lana School of Public Health and have varying levels of training and experience in qualitative methods. Faculty mentors SA, AA and SF have extensive experience in qualitative research, |  |
| 4. Gender | Was the researcher male or female? | Female – KB, MO, VA, MS, SA, AA, SF  Male – CD, DMV |  |
| 5. Experience and training | What experience or training did the researcher have? | Every group member has experience with qualitative research either through conducting structured or semi-structured interviews, or with conducting interview-based questionnaires in a variety of settings with different stakeholders. These included interviews with key informants, community leaders/members, and patients. | Under Materials and Methods |
| ***Relationship with participants*** | | | |
| 6. Relationship established | Was a relationship established prior to study commencement? | Potential participants were identified through publicly available information and the research team’s networks. | Under Materials and Methods |
| 7. Participant knowledge of the interviewer | What did the participants know about the researcher? e.g. personal goals, reasons for doing the research | An information sheet outlining the study’s objectives and accompanying consent form was shared with the interested informants prior to the interview. | Under Materials and Methods |
| 8. Interviewer characteristics | What characteristics were reported about the inter viewer/facilitator? e.g. Bias, assumptions, reasons and interests in the research topic | They both had awareness of the health systems in the provinces studied through reading literature and conducting the environmental scan. | Under Materials and Methods |

| **No. Item** | **Guide questions/description** | **Remarks** | **Reported on Page #** |
| --- | --- | --- | --- |
| **Domain 2: study design** | | | |
| ***Theoretical framework*** | | | |
| 9. Methodological orientation and Theory | What methodological orientation was stated to underpin the study? e.g. grounded theory, discourse analysis, ethnography, phenomenology, content analysis | An interpretive descriptive qualitative approach was used to analyze the qualitative interview data collected. | Under Materials and Methods |
| ***Participant selection*** | | | |
| 10. Sampling | How were participants selected? e.g. purposive, convenience, consecutive, snowball | Potential participants were identified through publicly available information and the research team’s networks. An information sheet outlining the study’s objectives and accompanying consent form was shared with the interested informants prior to the interview.  At the end of each interview, participants were asked if they had any suggestions as to who else we could interview to gather additional insights on the strategies used to deliver vaccines to our key populations and their implementation. Interviewees were also invited to provide additional resources on the strategies not captured in our initial environmental scan. Recruitment continued until data saturation was reached and no new themes emerged. | Under Materials and Methods |
| 11. Method of approach | How were participants approached? e.g. face-to-face, telephone, mail, email | Key informants across six selected Canadian provinces (Alberta, British Columbia, Manitoba, Nova Scotia, Ontario, and Quebec) were invited to participate in the study from November 2021 to April 2022 through email. | Under Materials and Methods |
| 12. Sample size | How many participants were in the study? | We conducted 25 individual and 6 group interviews (based on interviewees’ preference) with 41 key informants via Zoom (and, in one case, via email). Two key informants recused their interview data because of anonymity concerns, leading to 39 key informants’ data used in the current analysis. | Under Results |
| 13. Non-participation | How many people refused to participate or dropped out? Reasons? | Two key informants recused their interview data because of anonymity concerns, leading to 39 key informants’ data used in the current analysis. | Under Results |
| ***Setting*** | | | |
| 14. Setting of data collection | Where was the data collected? e.g. home, clinic, workplace | We conducted 25 individual and 6 group interviews (based on interviewees’ preference) with 41 key informants via Zoom (and, in one case, via email). | Under Results |
| 15. Presence of non-participants | Was anyone else present besides the participants and researchers? | No. | n/a |
| 16. Description of sample | What are the important characteristics of the sample? e.g. demographic data, date | The majority of participants were from Ontario, followed by Nova Scotia and Quebec (Figure 1). A total of 27 key informants (71%) represented an academic, government, or research institution, while 29% represented a community organization. About 1% of informants also served in national vaccine advisory boards. Around 90% of participants had prior experience with communicable disease control and vaccine safety and delivery. | Under results |
| ***Data collection*** | | | |
| 17. Interview guide | Were questions, prompts, guides provided by the authors? Was it pilot tested? | Moreover, respondents were asked questions similar to those displayed in Table 1, capturing the principles that guided the prioritization of populations for COVID-19 vaccination in their provinces and the strategies used to ensure adequate uptake of vaccines by the populations of interest in this study. Participants were also asked to reflect on the use of specific strategies and interventions and the factors that influence the implementation of the strategies.  Full interview guide with prompts will be attached. | Under Materials and Methods |
| 18. Repeat interviews | Were repeat interviews carried out? If yes, how many? | None were carried out | n/a |
| 19. Audio/visual recording | Did the research use audio or visual recording to collect the data? | Interviews were audio recorded on Zoom and transcripts were auto generated through Zoom's Audio transcript feature. A member of the research team listened to the audio to verify the accuracy of the transcript. | Under Materials and Methods |
| 20. Field notes | Were ﬁeld notes made during and/or after the interview or focus group? | Field notes were taken during the interview. | Under Materials and Methods |
| 21. Duration | What was the duration of the interviews or focus group? | Semi-structured interviews were led by two research team members (MS and VA) and averaged about 1 hour (interview length ranged from 33 to 85 minutes) | Under Materials and Methods |
| 22. Data saturation | Was data saturation discussed? | Recruitment continued until data saturation was reached and no new themes emerged. | Under Materials and Methods |
| 23. Transcripts returned | Were transcripts returned to participants for comment and/or correction? | Anonymized transcripts were also returned to key informants to be verified . | Under Materials and Methods |
| **Domain 3: analysis and ﬁndings** | | | |
| ***Data analysis*** | | | |
| 24. Number of data coders | How many data coders coded the data? | Data were analyzed independently by research team members (KB, MO, CD, DM) using inductive thematic analysis. | Under Materials and Methods |
| 25. Description of the coding tree | Did authors provide a description of the coding tree? | Yes | Under Materials and Methods |
| 26. Derivation of themes | Were themes identiﬁed in advance or derived from the data? | Creswell’s approach described | Under Materials and Methods |
| 27. Software | What software, if applicable, was used to manage the data? | The transcripts were imported into NVivo 12 software. | Under Materials and Methods |
| 28. Participant checking | Did participants provide feedback on the ﬁndings? | No | n/a |
| ***Reporting*** | | | |
| 29. Quotations presented | Were participant quotations presented to illustrate the themes/ﬁndings? Was each quotation identiﬁed? e.g. participant number | Yes | Under Results |
| 30. Data and ﬁndings consistent | Was there consistency between the data presented and the ﬁndings? | Yes | Under Results |
| 31. Clarity of major themes | Were major themes clearly presented in the ﬁndings? | Yes | Under Results |
| 32. Clarity of minor themes | Is there a description of diverse cases or discussion of minor themes? | Yes, all cases and themes were considered regardless of the number of times they appeared in the data | Under Results |
