# Supplementary material for: Strengthening access to and confidence in COVID-19 vaccines among equity-deserving populations across Canada: An exploratory qualitative study
Source: PLoS One. 2026 Apr 27;21(4):e0301953. doi: 10.1371/journal.pone.0301953 (PMC13120697; doi:10.1371/journal.pone.0301953)
Supplement: S2 Table — (DOCX) [file pone.0301953.s002.docx]

**S2 Table. Environmental scan data sources, search strategy and terms, eligibility criteria, and timeframe**

| **Data sources** | Provincial vaccination plans, federal and provincial media releases, reports from health research institutions and programs, media articles, academic and peer-reviewed publications |
| --- | --- |
| **Search Strategy** | - Targeted searches on the websites of provincial and federal government agencies, local health administrative authorities, community-based organizations, and health research institutions and program - Resources shared by subject-matter experts - Research databases (PubMed and Google Scholar) |
| **Search Terms** | Combination of keywords (including but not limited to):   - ***COVID-19 Vaccines***: "COVID-19" OR "SARS-CoV-2" OR "coronavirus"; “vaccine*" OR "immunization", OR "Pfizer" OR "Moderna" OR "Astra-Zeneca" - ***Reach***: "reach*" OR "access*" OR "deliver* OR "prioritize*" OR "uptake" OR "distribution" OR "roll-out" OR "equitable access" OR "availability" - ***Jurisdiction***: "Canada" OR "Alberta" OR "British Columbia" OR "Manitoba" OR "Nova Scotia" OR "Ontario" OR "Quebec" - ***Equity***: " "Health equity"; "Indigenous" OR "First Nations" OR "Métis" OR "FNIM"; "Black"; "BIPOC" OR "racialized communities"; "essential workers" OR "healthcare workers"; "homeless*"; "disability"; "social determinants of health" OR "socio-demographic*"; OR "underserved communities" OR "hard-to-reach populations" - ***Strategy for Vaccine Uptake and Confidence***: "policy" OR "program" OR "strategy*"; "community engagement" OR "stakeholder engagement" OR "partnership"; "communication"; "vaccination clinic"; "pharmacy"; "vaccine confidence" OR "vaccine acceptance"; "vaccine hesitancy" OR "vaccine mistrust" |
| **Inclusion Criteria** | - Material focusing on the access and delivery of a COVID-19 vaccine to the populations of interest (i.e., FNIM populations, Black communities, essential workers, people with disabilities, and people experiencing homelessness) - Material published between January 2020 and December 2021 - Material focusing on one of the Canadian provinces of interest (i.e., Alberta, British Columbia, Manitoba, Nova Scotia, Ontario, or Quebec) - Material published in English or French |
| **Exclusion Criteria** | - Material that does not focus on the access and delivery of a COVID-19 vaccine to at least one of the populations of interest. - Material published outside the specified timeframe (i.e., before January 2020 or after December 2021). - Material not focusing on one of the Canadian provinces of interest - Material not published in English or French. |
| **Timeframe** | - First environmental scan to include resources published between December 2020 and November 2021 - Second environmental scan to include previously missed and additional resources published between December 2020 and December 2021 |
