# Supplementary material for: Strengthening access to and confidence in COVID-19 vaccines among equity-deserving populations across Canada: An exploratory qualitative study
Source: PLoS One. 2026 Apr 27;21(4):e0301953. doi: 10.1371/journal.pone.0301953 (PMC13120697; doi:10.1371/journal.pone.0301953)
Supplement: S1 File — (DOCX) [file pone.0301953.s003.docx]

**S4 File. Key Informant Interview Guide**

Before the interview

1. introduce yourself and the project
2. ask participants whether they have received the information sheet, the consent form, and the provincial summary document
3. briefly recap the objectives of the study and the interview
4. ask participants whether they have any questions concerning the conduct of the interview
5. confirm that they are content with the interview being recorded
6. agree on a process to exchange the consent form (e.g., email)

**Questions:**

1. Can you briefly tell us about your role in the health system of [province]?

2. Can you tell us briefly about your role in implementing the COVID-19 vaccine in your province?

- What has your involvement been?
- What other COVID-related activities, outside your main role, have you been involved in?
- Do you have experience with other vaccine initiatives (e.g., the flu) before the COVID-19 pandemic?

3. Can you briefly speak to how the priority populations were selected in your province?

- Can you describe how decisions were made about prioritizing populations in your province?
- When you identified priority populations, was a framework used, and how closely was it followed?
- Was there any community representation on the advisory groups for population prioritization?

4. For this next question, this is going to be a multi-part question on each of the priority populations that we’re interested in. What barriers did the province face in terms of vaccine distribution and access for each of these populations? Are you aware of any strategies or interventions, if any, that were planned to reach or engage with the following populations to overcome these potential barriers to vaccine access and improve vaccine confidence? If yes, please share more details*.*

- First Nations, Inuit, and Métis populations
- Black communities
- Essential workers
- Individuals experiencing homelessness
- Individuals with disabilities

5. Do you think the correct priority groups were identified? In your opinion, why or why not?

- Was there any important population that was missed?
- Do you think the right approach was used to identify the priority groups?
- Were the groups prioritized in the right time period?

6. In your opinion, what did your provincial government do well in communicating the vaccine strategy to the public, including justifying why these populations were prioritized? What were opportunities for improvement?

7. We provided you with a summary document describing the vaccine rollout strategy as communicated by your province, focusing on the priority populations. Is the information accurate? Is there anything missing?

8. Do you know of any initiatives doing related work in this area (e.g., vaccine distribution among marginalized populations)?

- Who are the key stakeholders (individuals, organizations, etc.)?
- From your understanding, what is the work being done?

9. Is there anyone else you think we should speak to about the vaccine rollout?

- Is there anything else you would like to share?
- If we have any other questions, could we reach out?

**NACI-specific questions:**

1. Can you briefly tell us about your role within NACI?

2. Can you tell us briefly about your role in the COVID-19 vaccine implementation?

- What has your involvement been?
- What other COVID-related activities, outside your main role, have you been involved in?
- Do you have any previous experience with other vaccine initiatives (e.g., the flu) before the COVID-19 pandemic?

1. What was NACI’s approach to working with provinces in the roll-out? How closely did NACI work with provinces to define priority populations and discuss the implementation?

1. Was there a framework used by NACI, and if so, how closely was it followed?

- Did you look at the international context to help generate your recommendations? If so, which jurisdictions were studied/emulated, and why?
- Were there knowledge translation opportunities for learning from international jurisdictions?

1. Looking back, how would you assess NACI’s strategy of prioritizing the populations?

- Were the correct priority groups identified, or were any important populations overlooked in the NACI recommendations?
- Were priority groups identified in the right time period and/or order, or would you have altered anything?

1. For which recommendations did provinces deviate most from NACI’s guidelines, and which were closely followed by most provinces? Why do you think that is?

1. To your knowledge, are there best practices at the provincial level (including addressing vaccine barriers) for the equitable distribution of COVID-19 vaccines that NACI has used to update its recommendation guidelines or that NACI is looking to incorporate in its future guidance?
